# Characterisation of Natural Fibres for Sustainable Discontinuous Fibre Composite Materials

**DOI:** 10.3390/ma13092129

**Published:** 2020-05-04

**Authors:** Ali Kandemir, Thomas R. Pozegic, Ian Hamerton, Stephen J. Eichhorn, Marco L. Longana

**Affiliations:** Bristol Composites Institute (ACCIS), Department of Aerospace Engineering, School of Civil, Aerospace, and Mechanical Engineering, Queen’s Building, University of Bristol, University Walk, Bristol BS8 1TR, UK; tpozegic@yahoo.co.uk (T.R.P.); ian.hamerton@bristol.ac.uk (I.H.); s.j.eichhorn@bristol.ac.uk (S.J.E.); m.l.longana@bristol.ac.uk (M.L.L.)

**Keywords:** discontinuous fibre composites, fibre-matrix interfacial properties, mechanical properties, natural fibres

## Abstract

Growing environmental concerns and stringent waste-flow regulations make the development of sustainable composites a current industrial necessity. Natural fibre reinforcements are derived from renewable resources and are both cheap and biodegradable. When they are produced using eco-friendly, low hazard processes, then they can be considered as a sustainable source of fibrous reinforcement. Furthermore, their specific mechanical properties are comparable to commonly used, non-environmentally friendly glass-fibres. In this study, four types of abundant natural fibres (jute, kenaf, curaua, and flax) are investigated as naturally-derived constituents for high performance composites. Physical, thermal, and mechanical properties of the natural fibres are examined to evaluate their suitability as discontinuous reinforcements whilst also generating a database for material selection. Single fibre tensile and microbond tests were performed to obtain stiffness, strength, elongation, and interfacial shear strength of the fibres with an epoxy resin. Moreover, the critical fibre lengths of the natural fibres, which are important for defining the mechanical performances of discontinuous and short fibre composites, were calculated for the purpose of possible processing of highly aligned discontinuous fibres. This study is informative regarding the selection of the type and length of natural fibres for the subsequent production of discontinuous fibre composites.

## 1. Introduction

Owing to their light weight, superior specific strength, and stiffness, composite materials play a vital role in engineering applications and are continually replacing conventional monolithic materials. However, sustained growth in the composite industry can only be achieved if components and methodologies are sustainable, with the additional considerations of economic and environmental factors. Consequently, the composites industry is becoming increasingly aware of the importance of materials selection, manufacturing, end-of-life waste management strategies, and the life cycle assessment (LCA), a fundamental tool for the design phase [1,2].

Natural fibre reinforcements have attracted considerable attention in the composite industry owing to their specific mechanical properties and environmental advantages [3,4]. Natural fibres are an abundantly available, sustainable, biodegradable, and economically viable alternative to synthetic fibres, such as carbon, glass, and aramid, which share none of these characteristics [5]. Although natural fibres have begun to replace synthetic fibres in several applications [6], their use needs to be validated through comparison of their mechanical properties against those of reinforcements commonly used in industry. One of the drawbacks is the low thermal stability of natural fibres. This limits the possibility to couple them with high temperature processing polymeric matrices [4]. Another limiting factor that needs to be accounted for during the design and manufacturing is their hydrophilic nature; this is especially critical for applications wherein these materials are exposed to humid conditions [7].

Discontinuous fibres are easier to procure than continuous fibres, and their composites are able to exhibit high mechanical performances, comparable with those of continuous fibre counterparts if high levels of alignment and optimum critical fibre length are attained [8,9]. Moreover, highly aligned discontinuous fibre composites (ADFRC) have been considered as the best compromise where processability and performance requirements intersect [10]. The sustainability of composite materials in terms of manufacturing and recycling can be addressed by discontinuous fibre composite processing methods, such as the HiPerDiF method [11]. It has been shown that natural fibres can be manufactured by the HiPerDiF method, and hybrid flax/reclaimed carbon composites have exhibited significant cost reduction and increase in functional properties, i.e., damping, for the applications wherein a reduction in mechanical properties is an acceptable trade-off [12]. It is therefore possible to obtain a sustainable and reliable solution for high performance composites that use natural fibres in a discontinuous fibre composite processing method.

For obtaining high mechanical performances in discontinuous short fibre composites, one of the key parameters is the critical fibre length, which is highly dependent on the interfacial bonding between a matrix and a fibre. The interfacial shear strength (IFSS) is a measure of this interfacial bonding, and by using values of the IFSS, it is possible to calculate the critical fibre length. There are several methods with which to obtain IFSS [13]; the most common methods are fibre pull-out [14,15,16] and fibre fragmentation tests [17,18,19,20]. A third, the discrete approach, is the microbond test, which allows the direct and reproducible measurement of the single fibre-matrix interface strength, eliminating any meniscus effects [21,22], and has been widely preferred for evaluating the IFSS for different fibre/resin systems [23,24].

In this study, natural fibres, jute, kenaf, curaua, and flax were characterised to determine their physical, thermal, and mechanical properties, and these were compared with conventional synthetic fibres. The thermal stability of the fibres was determined using non-isothermal and isothermal thermogravimetric analysis (TGA) to ensure the operating temperature limit of the fibres. The tensile properties of the fibres were determined through single fibre tensile tests and the IFSS of the fibres with epoxy resin was obtained using the microbond test. The critical fibre length and aspect ratio of the fibres were calculated. These results are useful in informing the choices for fibre type and lengths that would be suitable for sustainable, high performance, discontinuous fibre composites.

## 2. Materials

Four promising natural fibres from different continents and climates—jute, kenaf, curaua, and flax, were used in this study. The jute fibres, provided from Shams UK Ltd., were Bangla Tossa fibres collected in Bangladeshi jute cultivation areas. The fibres were processed by the company by batching, carding, and drawing the filaments. The kenaf fibres, processed by a decorticator, were collected at Malang, East Java, Indonesia. The curaua fibres were collected at Santarem, Para, Brazil by conventional methods (hand crafted). The flax fibres, sourced from Eco-Technilin (Flaxtape™, Normandy, North of France) were produced using a proprietary process. All the fibres were off-the-shelf products and used as received; no sizing was present or applied to the fibres. Prior to testing, all fibres were dried in a vacuum oven at 70 °C, overnight, and samples were then stored in a desiccator to prevent further moisture uptake. In this study, the usage of the term *fibre* represents a bundle of ultimate fibres; i.e., fibrils, [25].

In this study, a commercially available epoxy resin, commonly used in industry to produce high performance composites, was selected. PRIME™20LV (*ex* Gurit) diamine-cured difunctional resin was used in combination with a PRIME™20 hardener with a weight ratio of 100:26. With a curing cycle of 7 h at 65 °C recommended by the manufacturer [26], this resin allows sufficient working time at room temperature to prepare the microbond test specimens.

## 3. Experimental Work

### 3.1. Physical Characterisation

#### 3.1.1. Visual Characterisation

**Figure 1:**
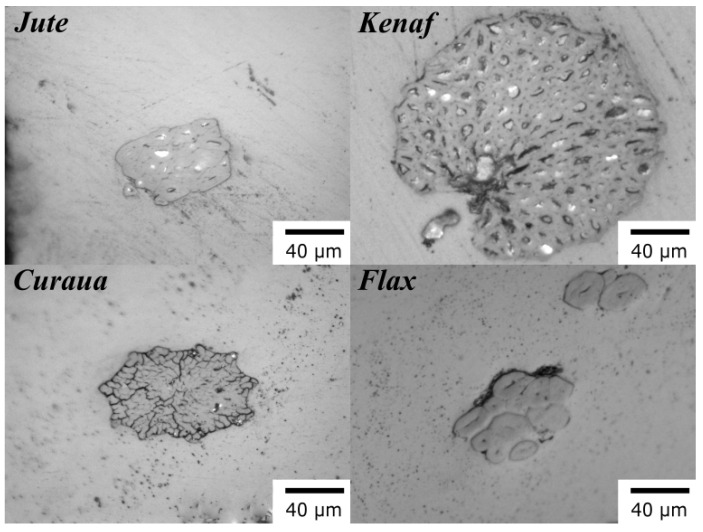
Cross-sectional optical microscopy images of jute, kenaf, curaua, and flax fibres.

Cold mounting was used to prepare the specimens for fibre cross-section examination under an optical microscope (Zeiss Axio Imager M2). Standard wet grinding and polishing for polymer matrix composites were used for each specimen. Figure 1 shows the cross-section optical microscopy images of the natural fibres. As seen in Figure 1, the cross-section of the kenaf fibres was found to be circular. For jute, the cross-sections were found to be more rectangular, and for flax more elliptical. Curaua fibres were found to have a circular cross-section with ragged edges. As also seen in Figure 1, kenaf fibres have considerably larger diameters than the other fibres, whereas the rest are of the same order of magnitude in terms of size. The diameter of each individual fibre bundle was determined using an optical microscope, taking an average at three points along the fibre; the measurements were performed on fifty specimens for each fibre type. For the post-processing of data in mechanical characterisation, fibres were assumed to be circular in their cross-sections. The diameters of the fibre bundles used in mechanical tests were measured to be 64.05 ± 5.93, 208.34 ± 12.08, 86.86 ± 2.39, and 63.76 ± 5.06 μm for jute, kenaf, curaua, and flax fibres, respectively.

#### 3.1.2. Density Characterisation

The apparent density, bulk density, apparent porosity, and water absorption of the natural fibres were obtained by following the principle of Archimedes (the buoyancy method) and recommendations of ASTM C830-00 using a precision balance (sensitivity 0.1 mg) [27]. Initially, each fibre was dried in a vacuum oven overnight and the dried fibre mass, *D*, was weighed immediately after removing from the oven. Each fibre was then immersed in water in a vacuum chamber, overnight, and the saturated weight of fibre, *W*, weighed in air afterwards. As a last step, the suspended weight of fibre, *S*, was measured while immersing the fibre in water.

Apparent (AD) and bulk density (BD) were calculated by using Equations (1) and (2), respectively:

(1) $$AD\;\left(g\;cm^{-3}\right)=\frac{D}{D-S}\times \rho_{\mathrm{medium}}$$

where $\rho_{\mathrm{medium}}$ denotes density of displacement medium.

(2) $$BD\;\left(g\;cm^{-3}\right)=\frac{D}{W-S}$$

Apparent porosity (AP) that describes open pores in terms of volume were calculated by Equation (3) as follows:

(3) $$AP\;\left(\%\right)=\frac{W-D}{W-S}\times 100$$

Water absorption (WA), which expresses the percentage of water absorbed by the dry fibre, was calculated by using Equation (4).

(4) $$WA\;\left(\%\right)=\frac{W-D}{D}\times 100$$

Table 1 shows the physical properties of the natural fibres. It was found that kenaf has the highest density (1.57 g cm^−3^) among them. The densities of jute, curaua, and flax were calculated to be 1.51, 1.50, and 1.54 g cm^−3^, respectively. The calculated density value for flax is in agreement with the literature values (1.54 [28] and 1.40–1.55 g cm^−3^ [29]), which were obtained from different methods, such as helium pycnometer (1.54 g cm^−3^ [30]), gas pycnometer (1.49–1.52 g cm^−3^ [31]), and immersion in water (1.54 g cm^−3^ [32]). The calculated density values for jute and curaua were found to be in close agreement with literature (1.30–1.50 g cm^−3^ [28,29] for jute; 1.52–1.56 g cm^−3^ [29] for curaua), whereas kenaf fibres (1.22–1.45 g cm^−3^ [29,33]) were found to be slightly higher. The bulk densities of the fibres were calculated to be 0.68, 0.78, 0.68, and 0.74 g cm^−3^ for jute, kenaf, curaua, and flax, respectively, displaying a correlation between apparent and bulk density. Moreover, it was seen that all fibres have the same significant amount of porosity within the range 50%–55%. On the contrary, the calculated water absorption values differ significantly. Curaua (∼82%) and jute (∼81%) fibres tend to absorb more water content compared to kenaf (∼65%) and flax (∼70%) fibres. It was concluded that the penetration of water into the natural fibres is considerable.

#### 3.1.3. Surface Analysis

Furthermore, Brunauer–Emmett–Teller (BET) surface area analysis of the natural fibre samples was performed in a static volumetric adsorption system (Micromeritics 3-Flex) using ultra-high pure N_2_ (Air Products. 99.9999%) up to 1 bar pressure. Before adsorption measurements, the samples, ∼150 g of chopped loose fibres, were heated up to 373 K under vacuum for 12 h to remove moisture and pre-adsorbed gases. The BET surface area was obtained within the relative pressure range of 0.05–0.25 at temperature 77 K.

Table 2 shows BET surface area measurements ($S_{BET}$) and calculated geometric surface area ($S_{geo}$), surface roughness (SR), and specific surface area (SSA) values of the fibres. Jute, which is processed by carding and drawing, showed the highest BET surface area, 2.28 m^2^g^−1^. It was found that kenaf and curaua have similar BET surface areas, 1.17 and 1.32 m^2^g^−1^, respectively. On the contrary, flax showed the lowest BET surface area, which is 0.37 m^2^g^−1^, similar to previously reported values (0.31–0.51 m^2^g^−1^ [34]). Additionally, BET surface areas of jute and curaua have been reported as 2.01 and 0.87 m^2^g^−1^ [35], which are consistent with the measured values.

To obtain better understanding about surface roughness, SR and SSA values (dependent and independent on diameter, respectively) were calculated. As seen in Table 2, it was found that flax fibres have higher surface roughness (9.05 ± 4.37) than glass fibres (∼1–1.7) [36]. However, jute, kenaf, and curaua fibres have nearly one order of magnitude higher surface roughness compared to flax fibres. The same trend was also observed in SSA values; flax fibres have the lowest SSA, while the other fibres have higher SSA values.

### 3.2. Thermal Analysis

The non-isothermal and isothermal decompositions were carried out in a simultaneous thermal analysis (STA) instrument (Netzsch STA 449 F1 Jupiter [Netzsch-Gerätebau GmbH, Wolverhampton, UK] ) under flowing nitrogen. The dynamic TGA data for the fibres were obtained between 40 and 800 °C at a heating rate of 10 °C min^−1^ and a nitrogen flow rate of 50 mL min^−1^. Two different temperatures, 175 °C and 225 °C, were maintained for 1 h to study the isothermal decomposition of the fibres under a nitrogen atmosphere with the same flow rate in the non-isothermal runs. Each sample weighed between 5 and 12 mg for each run of the decomposition tests and alumina crucibles were used as sample holders.

**Figure 2:**
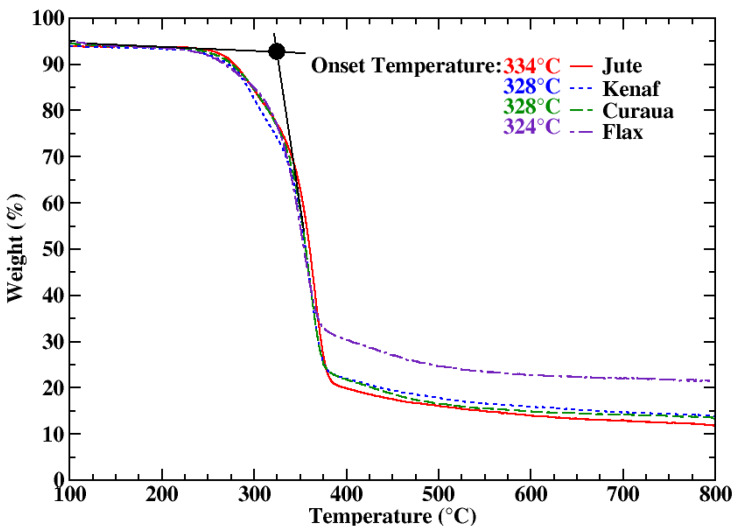
TGA curves of jute, kenaf, curaua, and flax fibres as a function of temperature. Onset temperatures of the fibres are shown as insets.

The dynamic TGA curves for natural fibres are shown in Figure 2. After initial weight loss caused by the vaporisation of absorbed moisture in the fibre, the first decomposition started at ∼220–230 °C and the onset temperature for the maximum decomposition, which is attributed to cellulose degradation in the structure [37], was found to be within 320–335 °C. Following the maximum decomposition, there is a gradual weight decrease related to lignin degradation, which requires higher condensation temperature [38]. Moreover, ∼80% weight loss was observed between the temperatures 150 and 700 °C for jute, kenaf, and curaua, whereas flax showed a lower mass loss (72%). Residual masses were ∼11.5%–13.5% for jute, kenaf, and curaua and ∼21% for flax at 800 °C.

**Figure 3:**
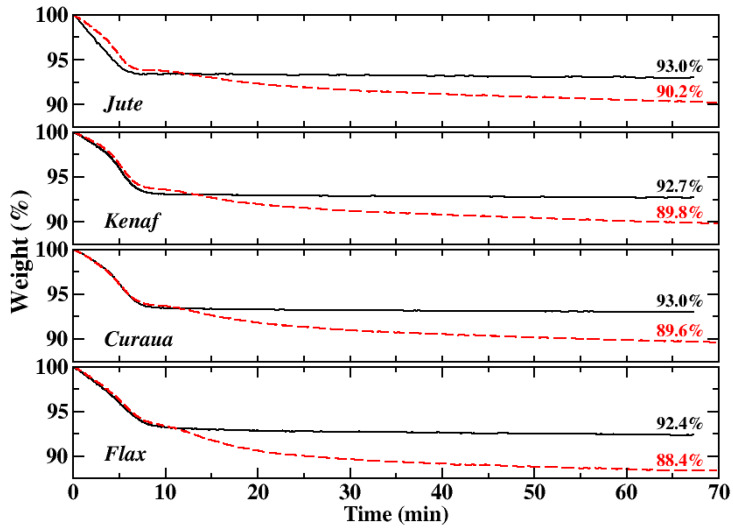
The isothermal TGA of jute, kenaf, curaua, and flax fibres during isothermal heating at 175 °C (black lines) and 225 °C (red dashed lines). Weights (%) after 1 h isothermal TGA run of the fibres are highlighted inside plots.

The dynamic TGA curves showed that significant decomposition starts at ∼220–230 °C for the fibres and as indicated for natural fibres [4]; they degrade after ∼200 °C. Therefore, isothermal TGA tests were carried out at 175 °C and at 225 °C; the isothermal TGA curves of the fibres are shown in Figure 3 (temperature profiles during tests as a function of time are also shown in the Supplementary Information; see Figure S6). The first mass loss, amounting to ∼7%, corresponding to water release from the fibres [37], occurring within 10 minutes during an isothermal analysis, was also observed in the dynamic TGA data (Figure 2). For 175 °C isothermal heating, no significant weight loss was observed for all fibres; gradual decrease in the weight of the fibres was seen during 225 °C isothermal heating. Thus, it was concluded that the fibres are thermally stable up to 175 °C.

### 3.3. Mechanical Characterisation

#### 3.3.1. Single Fibre Tensile Test

The mechanical properties of the natural fibres were determined by using a single fibre tensile test method (SFTT) [39]. The fibres were attached to plastic tabs that were arranged in a silicone holder to maintain a gauge length of 40 mm. Dynamax 3139 adhesive was used to attach the fibres to plastic tabs, which were subsequently cured under UV light for at least 2 h. Fibres underwent quasi-static tensile loading using a Dia-stron LEX820 Extensometer machine (Dia-Stron Limited, Andover, UK) using a 20 N load cell and with a strain rate of 0.02 mm/sec. For each fibre type, more than ten single fibres were successfully tested.

**Figure 4:**
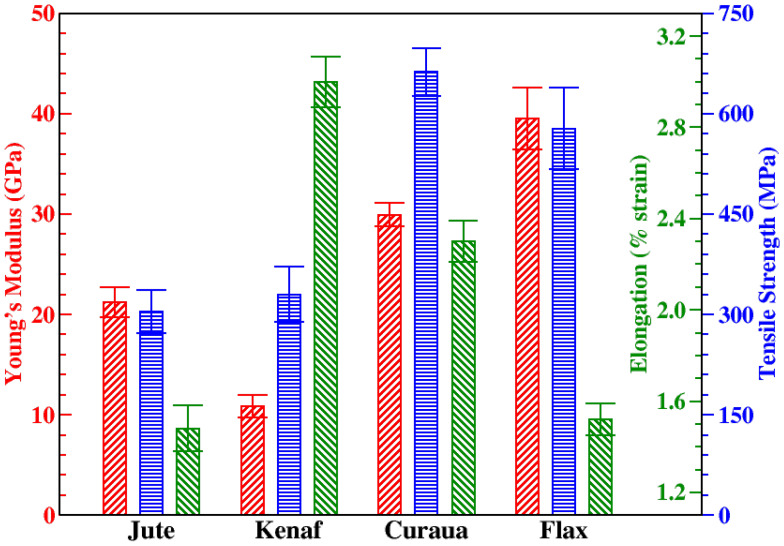
Young’s modulus (red /), tensile strength (blue −), and elongation to break (green \) values of jute, kenaf, curaua, and flax fibres.

Young’s moduli, tensile strength, and elongation at break of the fibres, which were assumed to have circular cross section, are shown in Figure 4. It was found that flax has the highest Young’s modulus (∼40 GPa) and curaua is the second highest (∼30 GPa) among the four fibres. Young’s modulus values for kenaf and jute were found to be ∼11 and 21 GPa, respectively. Moreover, curaua showed the highest tensile strength (∼660 MPa) and flax showed the second highest value (∼580 MPa). On the contrary, jute and kenaf showed similar lower tensile strength values, which are ∼300 and 330 MPa, respectively. In terms of stiffness and strength, the mechanical performances of curaua and flax fibres are better than those of jute and kenaf fibres. As expected, the stiffest fibre, i.e., flax, showed low elongation at break of 1.52 ± 0.07%; however, the lowest elongation at break was observed in jute—1.48 ± 0.10%, and could be related to the high content of cellulose compared to lignin in jute’s structure [40]. Owing to the high lignin content in kenaf fibres [41], their elongation at break was found to be 3.00 ± 0.11%, which is the highest amongst the other fibres. Curaua fibre has a moderate lignin content but also moderate hemicellulose and high cellulose contents in its structure [42], which might result high stiffness and moderate elongation at break (2.30 ± 0.09%).

Moreover, Weibull analysis was applied to the strength values of the fibres. Weibull analysis is a probabilistic approach and has been widely used to determine the statistical behaviour of the strengths of single fibres [43]. The Weibull shape (*m*) and scale parameter ($\sigma_{0}$) represent scatter of the data and the Weibull strength, respectively. The Weibull parameters for the natural fibres were calculated and $\sigma_{0}$ values of the fibres were found to be 341, 373, 717, and 646 MPa for jute, kenaf, curaua, and flax, respectively. It was seen that the Weibull strength values of the fibres are slightly higher than the mean strength values. *m* for jute, kenaf, curaua, and flax were calculated to be 2.98, 2.81, 5.88, and 3.06, respectively. Higher *m* means the low dispersion of Weibull strength, or in other words, fracture stress. It was seen that curaua has the highest *m* value, ∼6, and the other fibres show nearly same *m* value, ∼3.

#### 3.3.2. Microbond Test

**Figure 5:**
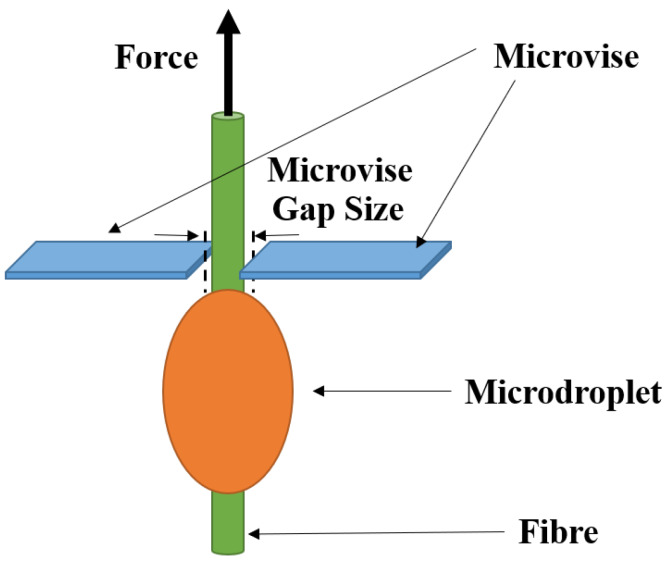
A schematic setup of the microbond test.

To determine the IFSS of the natural fibres with an epoxy matrix, a microbond method [21] was used. The microbond methodology followed the same methodology as the fibre tensile test preparation, initially; the fibres were attached to plastic tabs that were arranged in a silicone holder to maintain a gauge length of 40 mm. Dynamax 3139 adhesive was used to attach fibres to plastic tabs, which were subsequently cured at ambient temperature (ca. 20 °C) under UV light for at least 2 h. After this point, the epoxy resin droplets were applied to the fibres and cured in an oven following the manufacturers recommended procedure. An optical microscope was used to measure the droplet position on the fibre, the droplet size, and droplet embedded area for each microbond test. A Dia-stron LEX820 Extensometer was used with the microbond module which comprises a thin metallic plate (microvice) with a narrow cut in the middle to accommodate the fibre but prevent the microdroplet from passing through. A schematic of the microbond test setup is illustrated in Figure 5. Appropriate microvice gap separation (gap sizes; 50, 80, 150, 180, 225, 275, and 330 μm) was used depending on the size of the droplet to achieve a pure shear stress distribution. To determine the failure type, each fibre was observed using an optical microscope after the test.

**Figure 6:**
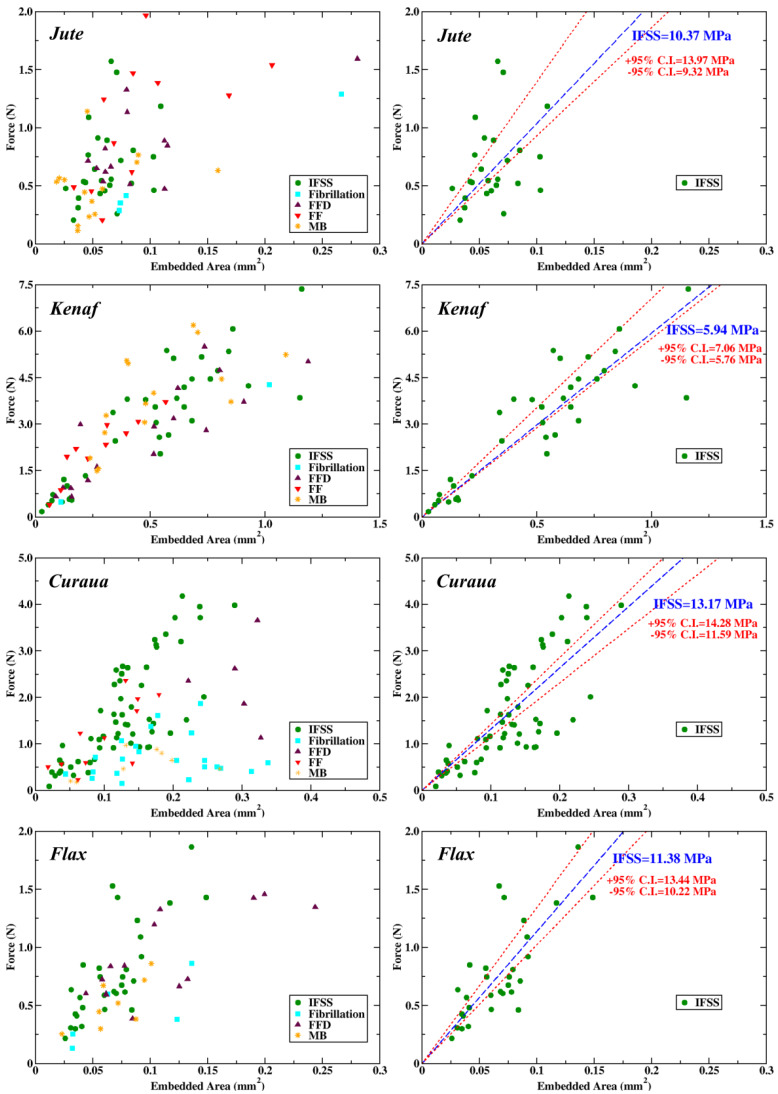
Microbond test results of jute, kenaf, curaua, and flax fibres in terms of debonding force versus embedded area. Left panel shows all data obtained from the test consist of different failure mechanisms. Right panel shows data only from samples that displayed a successful interfacial failure. Linear fit (blue dashed lines) was applied to the data, and results are shown in the figure with the upper and lower 95% confidence intervals (red dotted lines).

Figure 6 shows the microbond test results for the fibres as a function of debonding force and fibre embedded area. Different types of failure mechanisms, successful shear failure (IFSS), fibrillation of fibres within the droplet (fibrillation), fibre failure in the vicinity of the droplet (FFD), fibre failure (FF), and broken matrix (MB), were observed during the microbond tests (detail information of these failure types can be seen in the Supporting Information, see Figures S1–S5).

As seen in Figure 6, fibrillation failure takes place at low forces compared to other failure types for all fibres due to local weak interaction of ultimate fibres in a fibre bundle. No clear trend was seen for other failure types for jute and kenaf fibres. On the contrary, for curaua and flax it was seen that IFSS failure requires more force compared to other failure types, especially FFD and MB. The reason for this trend is related to the strengths of curaua and flax, which are stronger than jute and kenaf. It was also noted that mechanically stronger fibres are less likely to show fibre failure. For example, no fibre failure was observed for flax fibres and fewer fibre failures were observed for curaua fibres. Besides, it was seen that the shear distribution across the droplet, when it is contacted with the microvice, dictates the failure type. If the shear distribution is not equal at both sides of the droplet, generally, the failure tends to be FFD failure.

**Figure 7:**
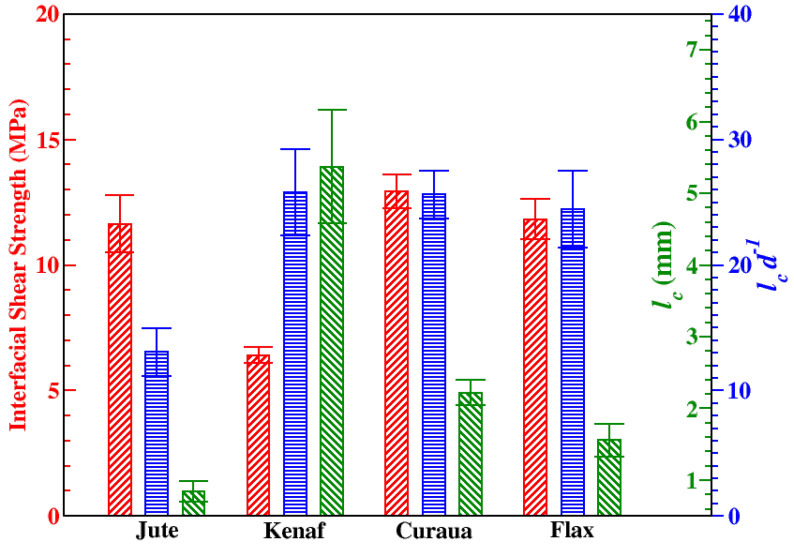
Interfacial shear strengths, IFSS (red /) and the critical fibre lengths, *l_c_* (green \), of jute, kenaf, curaua, and flax fibres; and *l_c_*/*d* (blue −) ratio values.

The IFSS between fibre and matrix is calculated with Equation (5):

(5) $$IFSS\left(MPa\right)=\frac{F_{d}\left(N\right)}{A_{e}\left(mm^{2}\right)}=\frac{F_{d}\left(N\right)}{\pi l_{e}d\left(mm^{2}\right)}$$

where *F_d_* and *A_e_* ($l_{e}$ and *d*) denote debonding force and embedded area (embedded length and fibre diameter), respectively. Applying a boundary condition Force(0)=0, a linear fit was applied to get an IFSS value for the fibres by considering only the successful shear failure samples, as shown in right panel of Figure 6. IFSS values obtained from linear fits are 10.37, 5.94, 13.17, and 11.38 MPa for jute, kenaf, curaua, and flax fibres, respectively. The mean and standard error of IFSS values were calculated to be 11.64 ± 1.13, 6.41 ± 0.32, 12.93 ± 0.67, and 11.83 ± 0.79 MPa for jute, kenaf, curaua, and flax fibres, respectively (Figure 7). As seen in Figure 6, IFSS values obtained from linear fits are within the range of upper and lower bounds of 95% confidence, and in fairly good agreement with the mean IFSS values.

To get effective reinforcement from ADFRC, the fibres must be longer than the critical fibre length, because this allows maximum load transfer amongst fibres and the failure of the composite material to be initiated by the fibres rather than fibre-matrix debonding. The critical fibre aspect ratio is calculated from following Equation (6) below.

(6) $$\frac{l_{c}}{d}=\frac{\sigma_{f}}{2\times IFSS}$$

where *l_c_*, *d*, and $\sigma_{f}$ denote the critical fibre length, diameter (at the droplet), and tensile strength of a fibre, respectively. Figure 7 shows the mean IFSS, *l_c_*, and *l_c_*/*d* ratio values of the fibres. Besides, the mechanical properties such as $\sigma_{f}$ and IFSS, *l_c_* depend on the fibre diameter, *d*, as seen in Equation (6). In our study, *l_c_* values of the fibres were found to be 0.84 ± 0.14, 5.37 ± 0.79, 2.22 ± 0.18, and 1.56 ± 0.23 mm for jute, kenaf, curaua, and flax, respectively. Since the diameters of the different fibres vary, it is worth reporting the ratio of *l_c_* to *d*, owing to the fact that it allows us to predict *l_c_* from the diameter. As seen in Figure 7, *l_c_*/*d* is similar (∼25) for kenaf, curaua, and flax; conversely, it is half of the value of those for jute, which shows the lowest *l_c_*.

## 4. Discussion

For composite manufacturing, the range over which the material is thermally stable defines the process parameters and the choice of matrix system. Having confirmed the necessity to pre-dry the fibres prior to processing, it was concluded that the natural fibres are stable up to ∼175 °C. This is a safe processing temperature for natural fibres since no significant decomposition was observed. It is also worth mentioning the fact that the drying processes are required to be able to process natural fibres with water-sensitive polymers and methods.

The IFSSs of jute, curaua, and flax fibres, were found to be approximately 11–14 MPa. Seghini et al., who used a similar epoxy (Prime 27 resin–Prime 20 hardener), calculated the IFSS with flax fibre through the single yarn fragmentation test, obtaining values between 16 and 24 MPa [18]. Furthermore, the IFSSs for flax with different epoxy systems have been reported as 33 MPa (24 MPa with Maleic anhydride sizing) [19] by using single fibre fragmentation tests, 23 MPa [44], and 13–17 MPa [14] by pull-out tests. It was noted that the obtained IFSS for flax fibre with our epoxy system is consistent with the lower bound of IFSS obtained by other research. On the other hand, there has been a large range of results for jute/epoxy from 4 MPa [45] obtained by using single fibre microbond test to 34–52 MPa [46] obtained by using single fibre pull-out tests. It is worth mentioning that the round-robin has also shown that pull-out tests give a higher IFSS value than the microbond tests [47]. To the best of the authors’ knowledge, there are no valid IFSS data for kenaf and curaua fibres with epoxy matrices available.

While the use of different epoxy systems prevents a direct comparison between the natural fibres and synthetic fibres, the natural fibres may show comparable IFSS values to those of glass fibres; however, the IFSS of glass fibres can be enhanced significantly by sizing. For instance, by using microbond tests, Baley et al. [23] showed that untreated flax/epoxy has an IFSS of 22.7 MPa, whereas glass with a textiloplastic sizing had a value of 29.3 MPa. Moreover, glass fibres that have different sizings, as coupling agents have exhibited IFSS values in the range 38–53 MPa with an epoxy matrix by using the microbond test [48]. Besides, it was found that carbon/epoxy has an IFSS value of 55–58 MPa with the microbond methodology [49], higher than the obtained IFSS values in this work and most of the reported values for natural fibres. There is no consensus for the effect of sizing on natural fibres to enhance or reduce IFSS due to the studies showing either enhancement or diminution [15,16,20]. However, it is also worth noting that natural fibres have a rougher surface, which enhances good mechanical interlocking with the matrix.

SR and SSA values revealed that jute, curaua, and flax fibres have different surface roughness characteristics that can be associated with IFSS values. As seen in Table 2, flax fibres have high IFSS values without significantly high SR and SSA values, whereas jute and curaua fibres have higher SR and SSA values which result in high IFSS values. SSA values of jute and curaua also show that jute fibres have more mechanical interlocking with the matrix per unit length compared to curaua. Since IFSS is a function of surface roughness, it can be concluded that flax has the highest performance with an epoxy matrix in terms of IFSS; however, jute and curaua fibres display better mechanical interlocking, which could be an important factor with another resin system.

Since some of the most important advantages of natural fibres are their specific mechanical properties, specific stiffnesses and strengths of the fibres were calculated by using measured density values. In addition to that, specific mechanical properties of the fibres are compared with conventional glass, carbon fibres, and other work in the literature, as shown in Table 3. The mechanical properties of natural fibres vary over a wide range; nevertheless, most of the obtained specific mechanical properties of the fibres are consistent with the literature. As seen in Table 3, the properties of flax fibres obtained in this work and the literature values of curaua and flax fibres are comparable with glass fibres.

In addition to that, some composite structural applications require high buckling performance, which is mainly related to the thickness of structures, and natural fibres have the advantage of being low density materials. Instead of using over-dimensioned conventional composite structures, natural fibre reinforced composite structures can provide high buckling performance without weight penalty. Additionally, it has been concluded that natural fibre reinforcements are preferable to glass reinforcements due to both the specific mechanical properties [52] and the environmental friendliness, as evidenced by LCA [53]. Furthermore, it is worth mentioning that the research performed by five different laboratories about the stiffness values of natural fibres has reported that Young’s modulus at low strain is 30% higher than the values obtained by SFTT [54]. Therefore, it can be concluded that natural fibres show higher Young’s moduli, and methods (such as SFTT) underestimate it.

### Natural Fibres as Alternatives to Glass Fibres in ADFRCs

By using the obtained data, it is possible to evaluate, in a hypothetical scenario, the effect of substituting glass with natural fibre in discontinuous fibre composites. A modified rule of mixtures, Equation (7), is used to compare the specific Young’s moduli of natural and glass fibre based ADFRCs:

(7) $$\frac{E_{c}}{\rho_{c}}=\eta_{0}\eta_{1}V_{f}\frac{E_{f}}{\rho_{f}}+V_{m}\frac{E_{m}}{\rho_{m}}$$

where $E_{c}$ is the highly aligned discontinuous fibre composite Young’s modulus in the fibre direction, $\rho_{c}$ is the density of the composite material, $\eta_{0}$ is a modulus reduction factor dependent on fibre orientation, $\eta_{1}$ is the fibre length efficiency factor, $E_{f}$ and $E_{m}$ are Young’s moduli of the fibre and matrix, and $\rho_{f}$ and $\rho_{m}$ are the densities of the fibre and matrix, respectively. For ADFRCs with high levels of fibre alignment, $\eta_{0}$ can be assumed to be ∼0.9, as shown by Yu et al. in [11]. $\eta_{1}$ can be calculated from the shear lag theory [55] with Equation (8),

(8) $$\eta_{1}=1-\frac{tanh(al/d)}{al/d}\;where\;a=\sqrt{\frac{-3E_{m}}{2E_{f}lnV_{f}}}$$

where *l* is the fibre length.

Owing to the superior mechanical properties compared to the natural fibres tested, flax and curaua fibres were selected for the case studies based on the hypothetical scenario of substituting glass with natural fibre in ADFRCs. Table 4 introduces the properties of fibres and matrices used in Equations (7) and (8) to obtain the mechanical properties of resultant ADFRCs. The properties of flax and curaua fibres are the ones obtained in this study corrected for SFTT underestimation [54].

**Figure 8:**
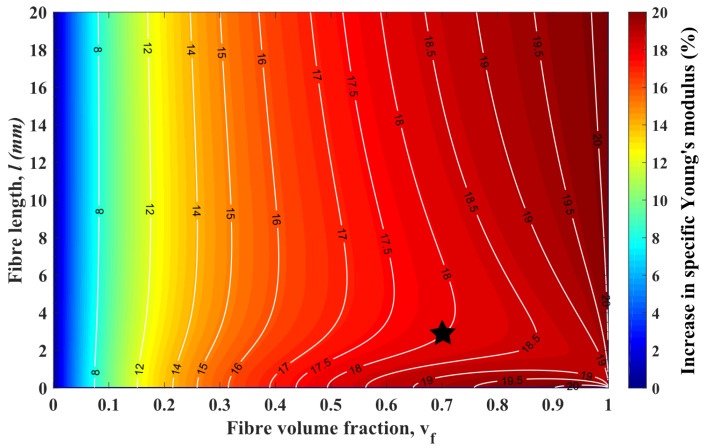
Contour plot of the increase in specific Young’s modulus, when highly aligned discontinuous glass fibre composites switched to highly aligned discontinuous flax fibre composites, in terms of fibre volume fraction and fibre length. White isolines are scaled by the right hand colour axis.

Figure 8 shows the contour plot of the increase in specific Young’s modulus (IiSYM) when flax fibre substitutes glass fibre in ADFRCs as a function of fibre length, *l*, and fibre volume fraction, $v_{f}$. In Figure 8, IiSYM increases from the dark blue to dark red regions and white lines evince the transition lines, which reveal the influence of *l* clearly.

As seen in Figure 8, there is more than 15% and up to 20% IiSYM in ADFRCs when glass fibres are substituted for flax fibres for every *l* and $v_{f}$ higher than 0.3. The effect of $v_{f}$ on IiSYM is constant and it leads to increase in IiSYM. On the contrary, *l* has two different effects on the IiSYM. From micron level to 1–10 mm, *l* leads to a dramatic decrease for IiSYM. After that, there is a gradual rise for IiSYM and for higher $v_{f}$; these trends get more clear. As an example, the black star in Figure 8 shows the condition of $v_{f}$ = 0.7 and *l* = 3 mm, which is higher than the $l_{c}$ of flax fibre, and it is the point at which 18% performance enhancement is seen. At that point, the effect of increasing *l* clearly shows two different trends, a significant decrease or a gradual increase.

**Figure 9:**
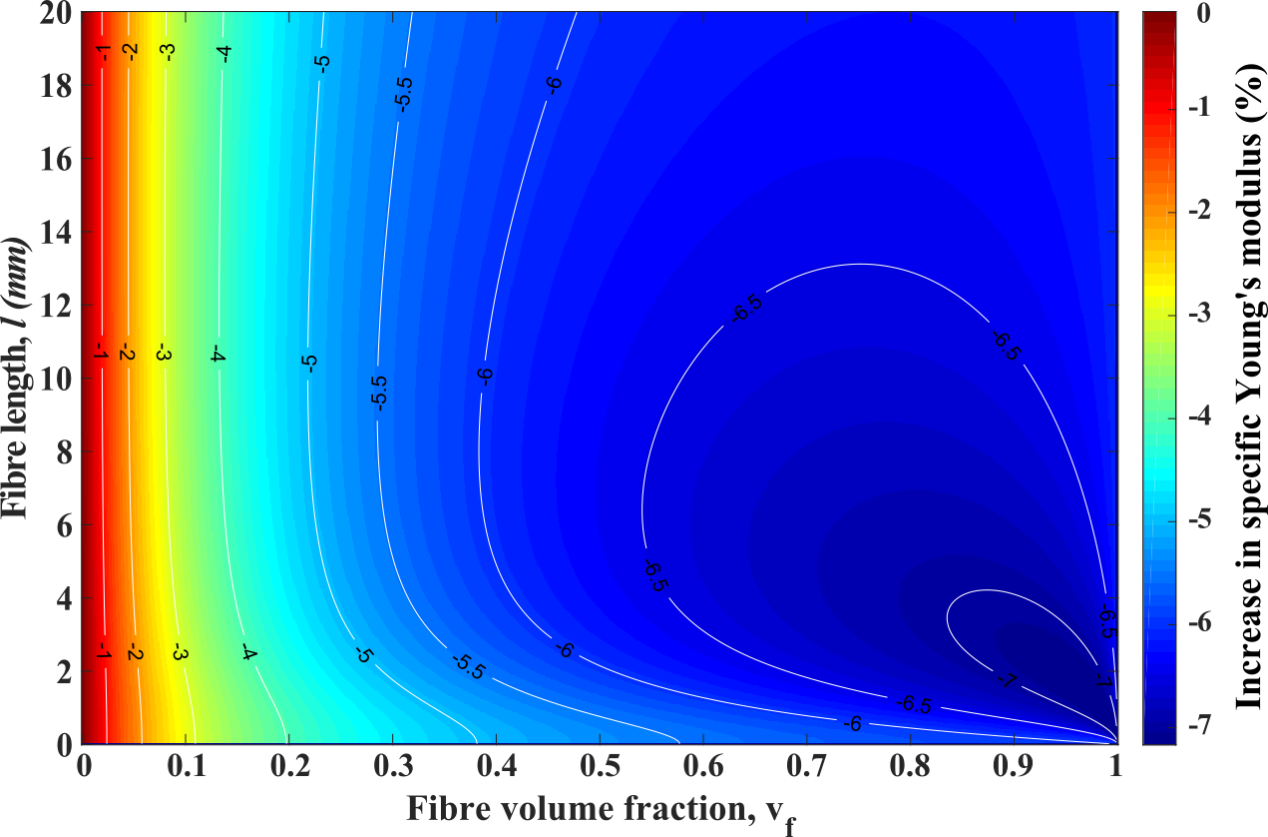
Contour plot of the increase in specific Young’s modulus, when highly aligned discontinuous glass fibre composites are switched to highly aligned discontinuous curaua fibre composites, in terms of fibre volume fraction and fibre length. White isolines are scaled by the right hand colour axis.

Moreover, the same hypothetical scenario was repeated for another promising fibre, curaua fibre, and Figure 9 shows the contour plot of the IiSYM when curaua fibre substitutes glass fibre in ADFRCs as a function of fibre length, and fibre volume fraction. It was seen that there is a ∼4.5%–6.5% decrease in specific Young’s modulus when $v_{f}$ is between 0.3 and 0.7 in the curaua fibre case. The same trend mentioned in the first case study about the IiSYM function of *l* was also seen clearly, especially at the bottom right corner of the plot; (i) first, IiSYM decreases when *l* increases from micron level to 1–2 mm; (ii) then IiSYM increases gradually. Even though curaua fibres in ADFRCs cause a decrease in mechanical properties when replacing glass fibres, advantages in environmental friendliness and sustainability remain and make the 5%–6% reduction in specific Young’s modulus an acceptable trade-off.

As expected, the stiffer flax fibre shows positive and better performance than curaua fibre in the hypothetical scenario of substituting glass with natural fibre in ADFRCs. However, curaua fibre shows agreeable reduction and may become more favourable than glass fibre if the sustainability aspects are taken into account through LCA as well.

## 5. Conclusions

In conclusion, considering the obtained physical, mechanical, interfacial, and thermal properties, jute, kenaf, curaua, and flax fibres were investigated as reinforcement candidates for sustainable discontinuous fibre composites. By using the mechanical, physical, and interfacial properties, the critical fibre lengths were also calculated to determine their reinforcement capabilities when used in the aligned discontinuous format. In addition, jute has the lowest critical fibre length value and the lowest critical aspect ratio among those tested. However, jute shows moderate mechanical properties compared to curaua and flax. In terms of mechanical and interfacial properties, curaua and flax were found to be promising natural fibre reinforcements to obtain high performance. It is foreseen that it will be possible to obtain more sustainable composites with specific mechanical properties comparable with those of glass fibre reinforced plastic.

Furthermore, thermogravimetric analysis based on the isothermal and non-isothermal TGA curves of the fibres revealed that the ideal processing temperature, where no significant degradation was observed, is ∼175 °C for the fibres. It was seen that the pre-dried fibres have moisture uptake approximately 6.5–7 wt.% from the environment, and during the heating period, the fibres lose all of the moisture uptake before the targeted temperature is reached.

From an experimental point of view, a better estimation of the embedded area by using a validated surface area with complementary high resolution microscope images would provide more information about the interfacial shear strengths of the natural fibres and the importance of mechanical interlocking at interface. Further steps include considering the interfacial properties between natural fibres and sustainable matrices (i.e., thermoplastics, bio-based resins, or covalently adaptable networks) to improve the sustainability of natural fibre reinforced composites. Thereafter, the complete set of data should be mapped and allow one to find the junction point of high performance and sustainable composites for ADFRCs.

This study allows sustainable material selection as reinforcement for ADFRC, and the prime candidates were determined to be curaua and flax fibres. It was also demonstrated that flax fibres perform better mechanically compared to glass fibres in ADFRCs. The potential applications would be the replacement of unsustainable composites with sustainable ADFRCs in engineering applications, such as sporting goods, transport, and automobile industries in which weight reduction and sustainability are of paramount importance.

## Figures and Tables

**Table 1:** Physical properties of jute, kenaf, curaua, and flax fibres. Errors represent standard errors of means (SEM).

Fibre	Apparent	Apparent	Water	Bulk
	Density	Porosity	Absorption	Density
	(g cm^−3^)	(%)	(%)	(g cm^−3^)
Jute	1.51 ± 0.01	54.86 ± 2.20	81.08 ± 7.75	0.68 ± 0.04
Kenaf	1.57 ± 0.02	50.53 ± 1.20	65.21 ± 3.76	0.78 ± 0.03
Curaua	1.50 ± 0.01	54.58 ± 5.61	81.98 ± 17.57	0.68 ± 0.08
Flax	1.54 ± 0.01	51.72 ± 0.15	69.60 ± 0.19	0.74 ± 0.00

**Table 2:** Surface area properties of jute, kenaf, curaua, and flax fibres. S*_BET_*, S*_geo_*, SR, and SSA denote BET surface area, geometric surface area, surface roughness, and specific surface area, respectively. Errors represent SEM. ($S_{geo}=4{\left(\rho d\right)}^{-1},\;SR=S_{BET}/S_{geo},\;SSA=\rho S_{BET}$).

Fibre	S*_BET_*	S*_geo_*	SR	SSA
	(m^2^ g^−1^)	10^−2^ (m^2^ g^−1^)		(μm^−1^)
Jute	2.28 ± 1.07	4.14 ± 0.38	55.11 ± 26.35	3.44 ± 1.61
Kenaf	1.17 ± 0.12	1.22 ± 0.07	96.04 ± 11.36	1.84 ± 0.19
Curaua	1.32 ± 0.61	3.07 ± 0.09	43.02 ± 19.97	1.98 ± 0.92
Flax	0.37 ± 0.18	4.07 ± 0.32	9.05 ± 4.37	0.57 ± 0.27

**Table 3:** Specific mechanical properties of jute, kenaf, curaua, and flax fibres compared with literature values of natural and synthetic fibres. Errors represent SEMs.

	Specific Young’s Modulus	Specific Strength	Failure
This Study	(GPa cm^3^ g^−1^)	(MPa cm^3^ g^−1^)	Strain (%)
Jute	14.04 ± 1.00	201.36 ± 21.52	1.48 ± 0.10
Kenaf	6.93 ± 0.71	210.52 ± 26.45	3.00 ± 0.11
Curaua	19.96 ± 0.77	441.53 ± 24.07	2.30 ± 0.09
Flax	25.64 ± 2.01	375.36 ± 39.72	1.52 ± 0.07
Glass [50]	28–30	940–1350	2.5–3.4
Jute [50]	7–39	270–650	1.2–2.0
Kenaf [50]	12–42	538	3.0
Curaua [50]	8.4–36	360–1000	3.0–4.3
Flax [50]	26–76	240–1070	1.2–3.3
Carbon [51]	128–130	1900–2700	1.5–2.1

**Table 4:** Material properties used in the case studies.

Parameters	Natural Fibres		Glass Fibre	Matrix
	Flax	Curaua	C100, Vetrotex	PRIME™20LV
			[56]	[26]
*E* (GPa)	52	39	73	3.50
*ρ* (g cm^−3^)	1.54	1.50	2.60	1.15
*d* (mm)	64	87	7	-

## Supplementary Materials

The following are available online at https://www.mdpi.com/1996-1944/13/9/2129/s1. All underlying data are provided in this published article (and its supplementary information files).

## References

[1] U.K. Composites (2015). End-of-Life Solutions for FRP Composites.

[2] Tapper R.J., Longana M.L., Norton A., Potter K.D., Hamerton I. (2020). An evaluation of life cycle assessment and its application to the closed-loop recycling of carbon fibre reinforced polymers. Compos. Part B Eng..

[3] Pickering K., Efendy M.A., Le T. (2016). A review of recent developments in natural fibre composites and their mechanical performance. Compos. Part A Appl. Sci. Manuf..

[4] Summerscales J., Dissanayake N.P., Virk A.S., Hall W. (2010). A review of bast fibres and their composites. Part 1—Fibres as reinforcements. Compos. Part A Appl. Sci. Manuf..

[5] Saheb D.N., Jog J.P. (1999). Natural fiber polymer composites: A review. Adv. Polym. Technol..

[6] Fangueiro R., Rana S. (2016). Natural Fibres: Advances in Science and Technology Towards Industrial Applications From Science to Market.

[7] Wang W., Sain M., Cooper P. (2006). Study of moisture absorption in natural fiber plastic composites. Compos. Sci. Technol..

[8] Fukuda H., Chou T.W. (1982). A probabilistic theory of the strength of short-fibre composites with variable fibre length and orientation. J. Mater. Sci..

[9] Fu S.Y., Lauke B. (1996). Effects of fiber length and fiber orientation distributions on the tensile strength of short-fiber-reinforced polymers. Compos. Sci. Technol..

[10] Matthew Such C.W., Potter K. (2014). Aligned Discontinuous fibre composites: A short history. J. Multifunct. Compos..

[11] Yu H., Potter K., Wisnom M. (2014). A novel manufacturing method for aligned discontinuous fibre composites (High Performance-Discontinuous Fibre method). Compos. Part A Appl. Sci. Manuf..

[12] Longana M.L., Ondra V., Yu H., Potter K.D., Hamerton I. (2018). Reclaimed Carbon and Flax Fibre Composites: Manufacturing and Mechanical Properties. Recycling.

[13] Drzal L., Madhukar M. (1993). Fibre-matrix adhesion and its relationship to composite mechanical properties. J. Mater. Sci..

[14] Spārniņš E., Nyström B., Andersons J. (2012). Interfacial shear strength of flax fibers in thermoset resins evaluated via tensile tests of UD composites. Int. J. Adhes. Adhes..

[15] Wong S., Shanks R., Hodzic A. (2007). Effect of additives on the interfacial strength of poly(l-lactic acid) and poly(3-hydroxy butyric acid)-flax fibre composites. Compos. Sci. Technol..

[16] Arbelaiz A., Cantero G., Fernández B., Mondragon I., Gañán P., Kenny J. (2005). Flax fiber surface modifications: Effects on fiber physico mechanical and flax/polypropylene interface properties. Polym. Compos..

[17] Zafeiropoulos N., Baillie C., Hodgkinson J. (2002). Engineering and characterisation of the interface in flax fibre/polypropylene composite materials. Part II. The effect of surface treatments on the interface. Compos. Part A Appl. Sci. Manuf..

[18] Seghini M., Touchard F., Sarasini F., Chocinski-Arnault L., Mellier D., Tirillò J. (2018). Interfacial adhesion assessment in flax/epoxy and in flax/vinylester composites by single yarn fragmentation test: Correlation with micro-CT analysis. Compos. Part A Appl. Sci. Manuf..

[19] Joffe R., Andersons J., Wallström L. (2005). Interfacial shear strength of flax fiber/thermoset polymers estimated by fiber fragmentation tests. J. Mater. Sci..

[20] Huber T., Müssig J. (2008). Fibre matrix adhesion of natural fibres cotton, flax and hemp in polymeric matrices analyzed with the single fibre fragmentation test. Compos. Interfaces.

[21] Gaur U., Miller B. (1989). Microbond method for determination of the shear strength of a fiber/resin interface: Evaluation of experimental parameters. Compos. Sci. Technol..

[22] Khalil H., Ismail H., Rozman H., Ahmad M. (2001). The effect of acetylation on interfacial shear strength between plant fibres and various matrices. Eur. Polym. J..

[23] Baley C., Busnel F., Grohens Y., Sire O. (2006). Influence of chemical treatments on surface properties and adhesion of flax fibre–polyester resin. Compos. Part A Appl. Sci. Manuf..

[24] Wang F., Lu M., Zhou S., Lu Z., Ran S. (2019). Effect of Fiber Surface Modification on the Interfacial Adhesion and Thermo-Mechanical Performance of Unidirectional Epoxy-Based Composites Reinforced with Bamboo Fibers. Molecules.

[25] Vincent J.F.V. (2000). A Unified Nomenclature for Plant Fibres for Industrial Use. Appl. Compos. Mater..

[26] (2019). Gurit, Prime™20lv Datasheet.

[27] ASTM International (2016). Standard Test Methods for Apparent Porosity, Liquid Absorption, Apparent Specific Gravity, and Bulk Density of Refractory Shapes by Vacuum Pressure.

[28] Cook J.G. (2001). Handbook of Textile Fibres: Volume 1: Natural Fibres.

[29] Lewin M. (2010). Handbook of Fiber Chemistry.

[30] Sawsen C., Fouzia K., Mohamed B., Moussa G. (2014). Optimizing the formulation of flax fiber-reinforced cement composites. Constr. Build. Mater..

[31] Amiri A., Triplett Z., Moreira A., Brezinka N., Alcock M., Ulven C.A. (2017). Standard density measurement method development for flax fiber. Ind. Crop. Prod..

[32] Madsen B., Lilholt H. (2003). Physical and mechanical properties of unidirectional plant fibre composites—An evaluation of the influence of porosity. Compos. Sci. Technol..

[33] Bourmaud A., Beaugrand J., Shah D.U., Placet V., Baley C. (2018). Towards the design of high-performance plant fibre composites. Prog. Mater. Sci..

[34] Bismarck A., Aranberri-Askargorta I., Springer J., Lampke T., Wielage B., Stamboulis A., Shenderovich I., Limbach H.H. (2002). Surface characterization of flax, hemp and cellulose fibers; Surface properties and the water uptake behavior. Polym. Compos..

[35] Cordeiro N., Gouveia C., Moraes A., Amico S. (2011). Natural fibers characterization by inverse gas chromatography. Carbohydr. Polym..

[36] Wesson S.P., Vajo J.J., Ross S. (1983). Determination of specific surface areas of glass filaments by BET and CAEDMON methods. J. Colloid Interface Sci..

[37] Spinacé M.A., Lambert C.S., Fermoselli K.K., Paoli M.A.D. (2009). Characterization of lignocellulosic curaua fibres. Carbohydr. Polym..

[38] Ramiah M. (1970). Thermogravimetric and differential thermal analysis of cellulose, hemicellulose, and lignin. J. Appl. Polym. Sci..

[39] ASTM International (2014). Standard Test Method for Tensile Strength and Young’s Modulus of Fibers.

[40] Komuraiah A., Kumar N.S., Prasad B.D. (2014). Chemical Composition of Natural Fibers and its Influence on their Mechanical Properties. Mech. Compos. Mater..

[41] Mortazavi S.M., Kamali Moghaddam M. (2010). An analysis of structure and properties of a natural cellulosic fiber (Leafiran). Fibers Polym..

[42] Ouajai S., Shanks R. (2005). Composition, structure and thermal degradation of hemp cellulose after chemical treatments. Polym. Degrad. Stab..

[43] Swolfs Y., Verpoest I., Gorbatikh L. (2016). A review of input data and modelling assumptions in longitudinal strength models for unidirectional fibre-reinforced composites. Compos. Struct..

[44] Eichhorn S.J., Baillie C.A., Zafeiropoulos N., Mwaikambo L.Y., Ansell M.P., Dufresne A., Entwistle K.M., Herrera-Franco P.J., Escamilla G.C., Groom L. (2001). Review: Current international research into cellulosic fibres and composites. J. Mater. Sci..

[45] Sarker F., Karim N., Afroj S., Koncherry V., Novoselov K.S., Potluri P. (2018). High-Performance Graphene-Based Natural Fiber Composites. ACS Appl. Mater. Interfaces.

[46] Doan T.T.L., Brodowsky H., Mäder E. (2012). Jute fibre/epoxy composites: Surface properties and interfacial adhesion. Compos. Sci. Technol..

[47] Pitkethly M.J., Favre J.P., Gaur U., Jakubowski J., Mudrich S.F., Caldwell D.L., Drzal L.T., Nardin M., Wagner H.D., Di Landro L. (1993). A round-robin programme on interfacial test methods. Compos. Sci. Technol..

[48] Bryce D., Yang L., Thomason J. An investigation of fibre sizing on the interfacial strength of glass-fibre epoxy composites. Proceedings of the ECCM18—18th European Conference on Composite Materials.

[49] Kang S.K., Lee D.B., Choi N.S. (2009). Fiber/epoxy interfacial shear strength measured by the microdroplet test. Compos. Sci. Technol..

[50] Charlet K. (2012). CHAPTER 3 Natural Fibres as Composite Reinforcement Materials: Description and New Sources. Natl. Polym..

[51] (2019). Toray Carbon Fibres, Torayca T300 and Toray T700s Technical Data Sheet.

[52] Wambua P., Ivens J., Verpoest I. (2003). Natural fibres: Can they replace glass in fibre reinforced plastics?. Compos. Sci. Technol..

[53] Correa J.P., Montalvo-Navarrete J.M., Hidalgo-Salazar M.A. (2019). Carbon footprint considerations for biocomposite materials for sustainable products: A review. J. Clean. Prod..

[54] Bensadoun F., Verpoest I., Baets J., Müssig J., Graupner N., Davies P., Gomina M., Kervoelen A., Baley C. (2017). Impregnated fibre bundle test for natural fibres used in composites. J. Reinf. Plast. Compos..

[55] Cox H.L. (1952). The elasticity and strength of paper and other fibrous materials. Br. J. Appl. Phys..

[56] Yu H., Longana M.L., Jalalvand M., Wisnom M.R., Potter K.D. (2018). Hierarchical pseudo-ductile hybrid composites combining continuous and highly aligned discontinuous fibres. Compos. Part A Appl. Sci. Manuf..

