# Supplementary material for: Characterisation of Natural Fibres for Sustainable Discontinuous Fibre Composite Materials
Source: Materials (Basel). 2020 May 4;13(9):2129. doi: 10.3390/ma13092129 (PMC7254363; doi:10.3390/ma13092129)
Supplement: Supplementary file 1 [file materials-13-02129-s001.pdf]

Supporting information to

Characterisation of natural fibres for sustainable discontinuous fibre composite materials

Ali Kandemir*, Thomas R. Pozegic, Ian Hamerton, Steve J. Eichhorn, Marco L. Longana
Bristol Composites Institute (ACCIS), Department of Aerospace Engineering, University of Bristol, Bristol, BS8 1TR, UK
e-mail address: ali.kandemir@bristol.ac.uk

Failure types during IFSS test

Following figures (S1-S5) show examples of the failure types (IFSS, Fibrillation, FFD, FF, and MB) observed in IFSS tests. It is important to check droplet position before test to decide failure type, especially for IFSS since it may be deceptive to check only test data.

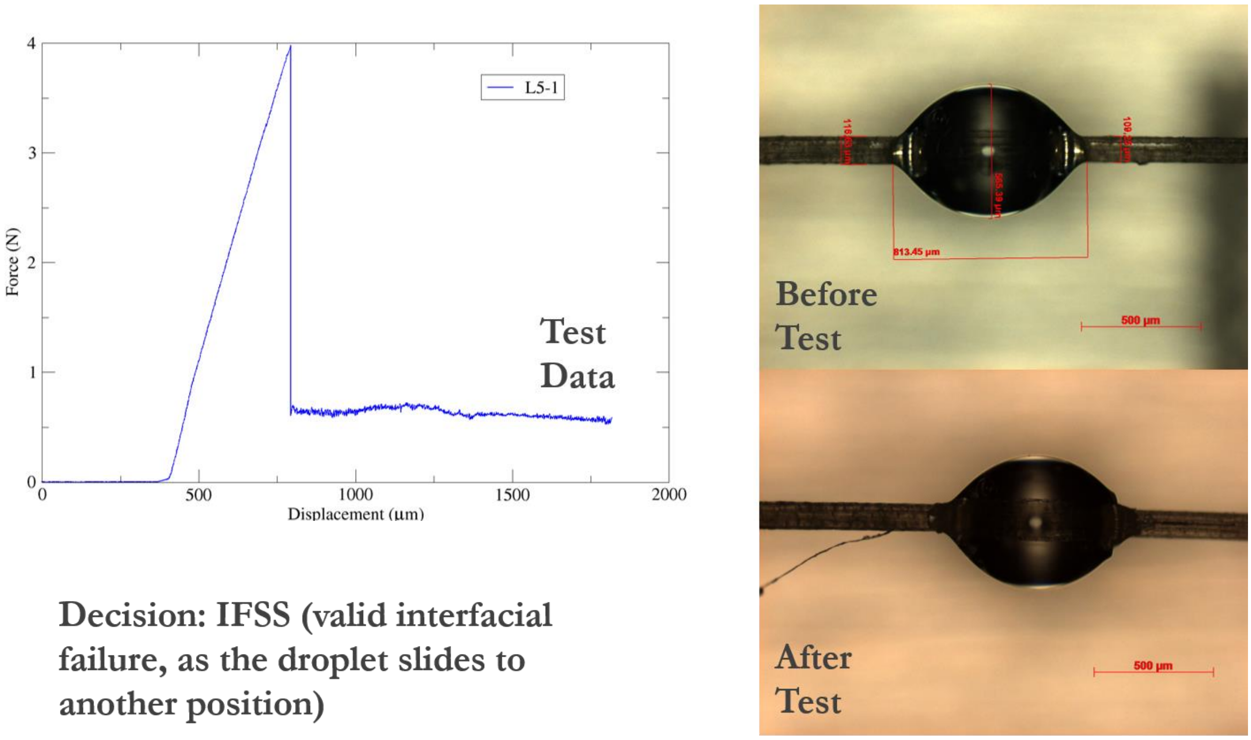

Figure S1. IFSS failure.

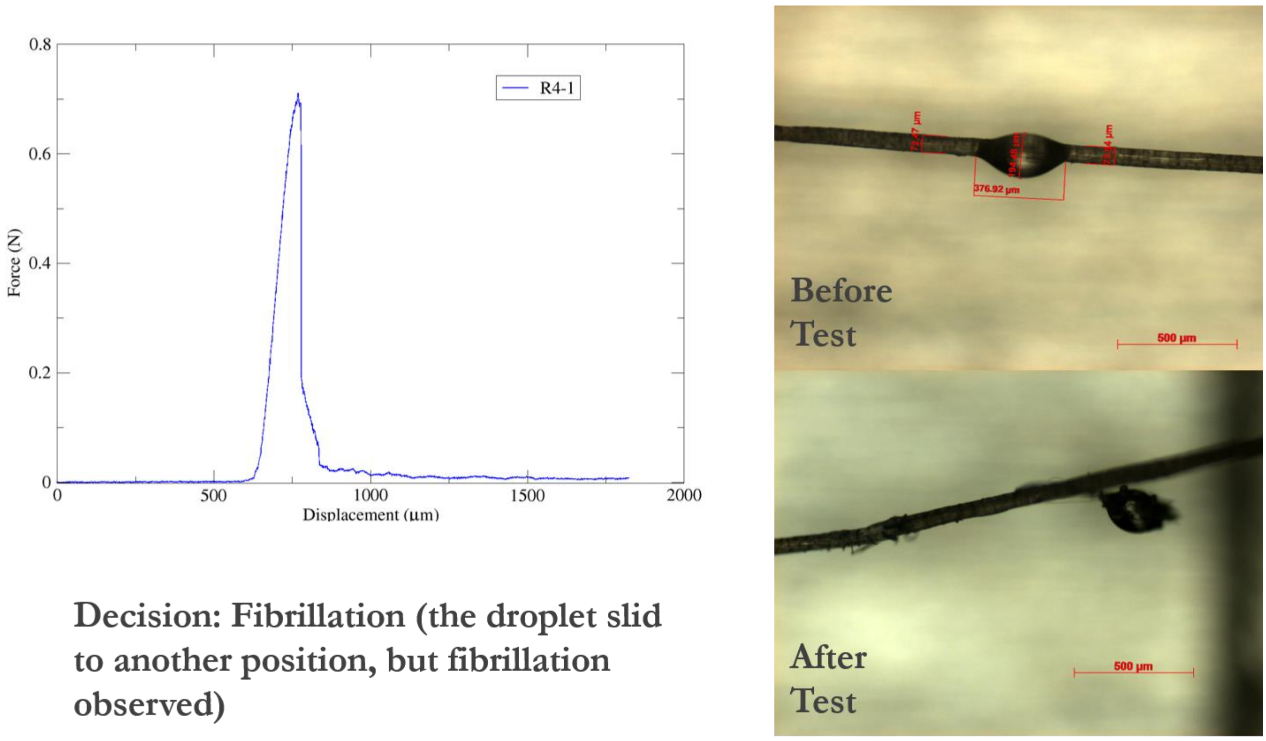

Figure S2. Fibrillation failure.

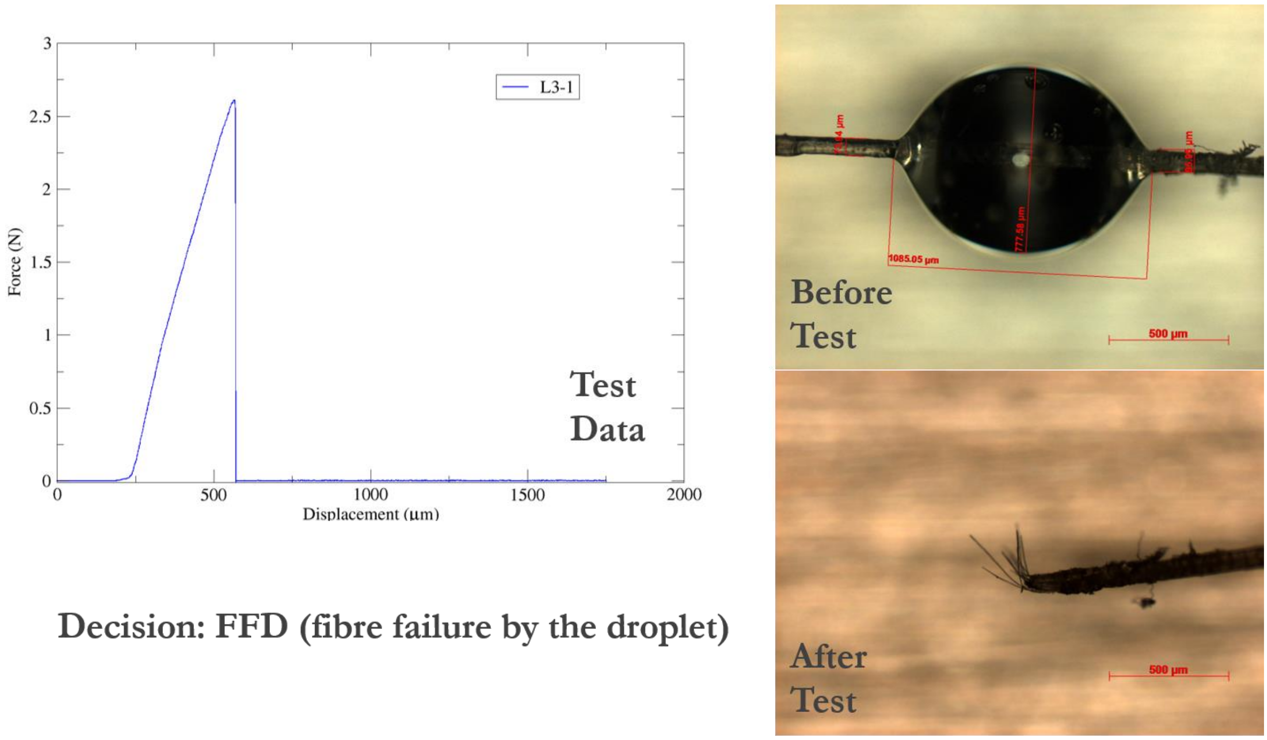

Figure S3. FFD failure.

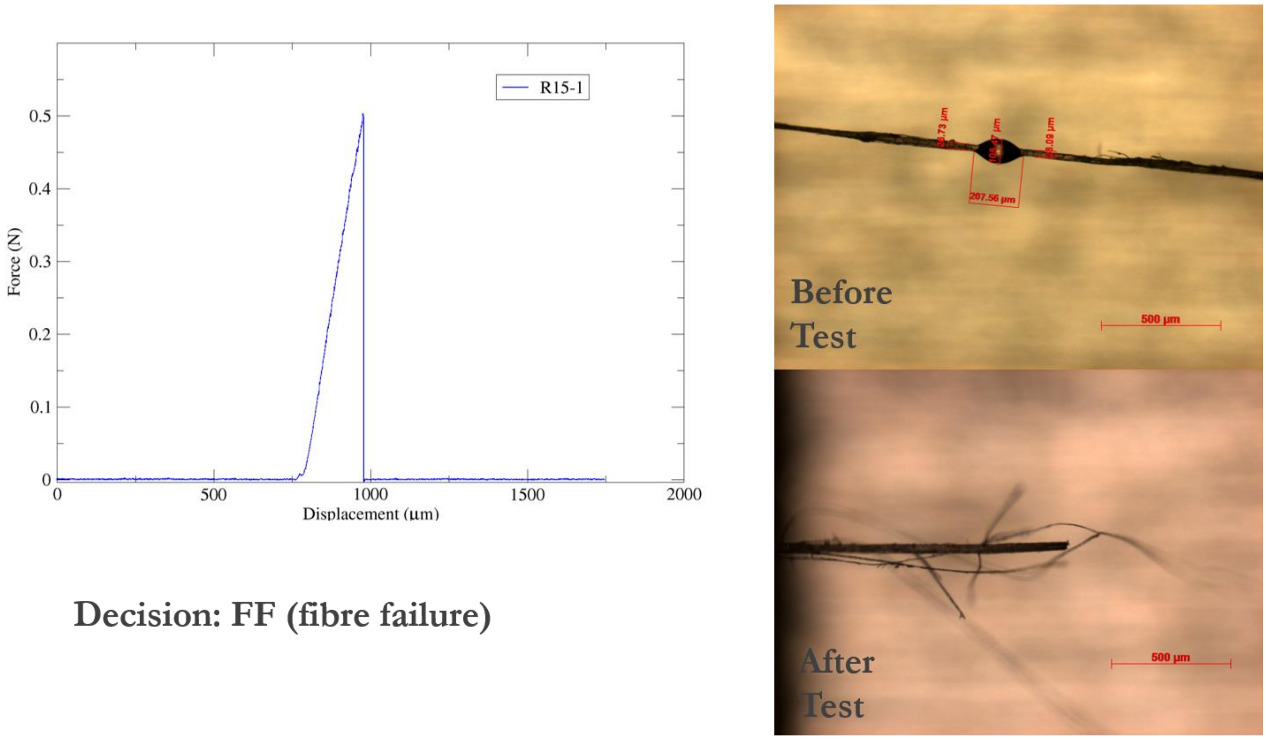

Figure S4. FF failure.

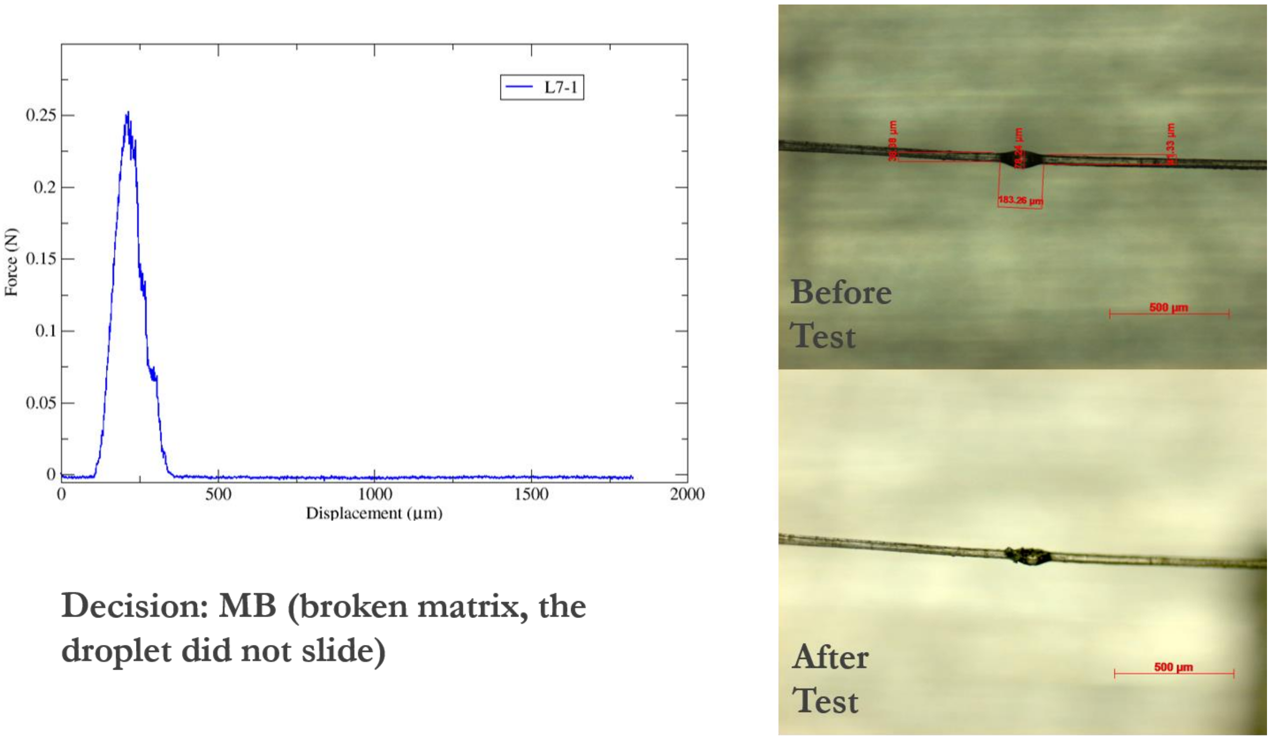

Figure S5. MB failure.

The isothermal TGA test

Figure S6 shows temperature profile during the isothermal TGAs. As seen in the figure, +10°C temperature strike happened during the tests and the fibres were exposed to over temperature until the last 30 minutes of the tests.

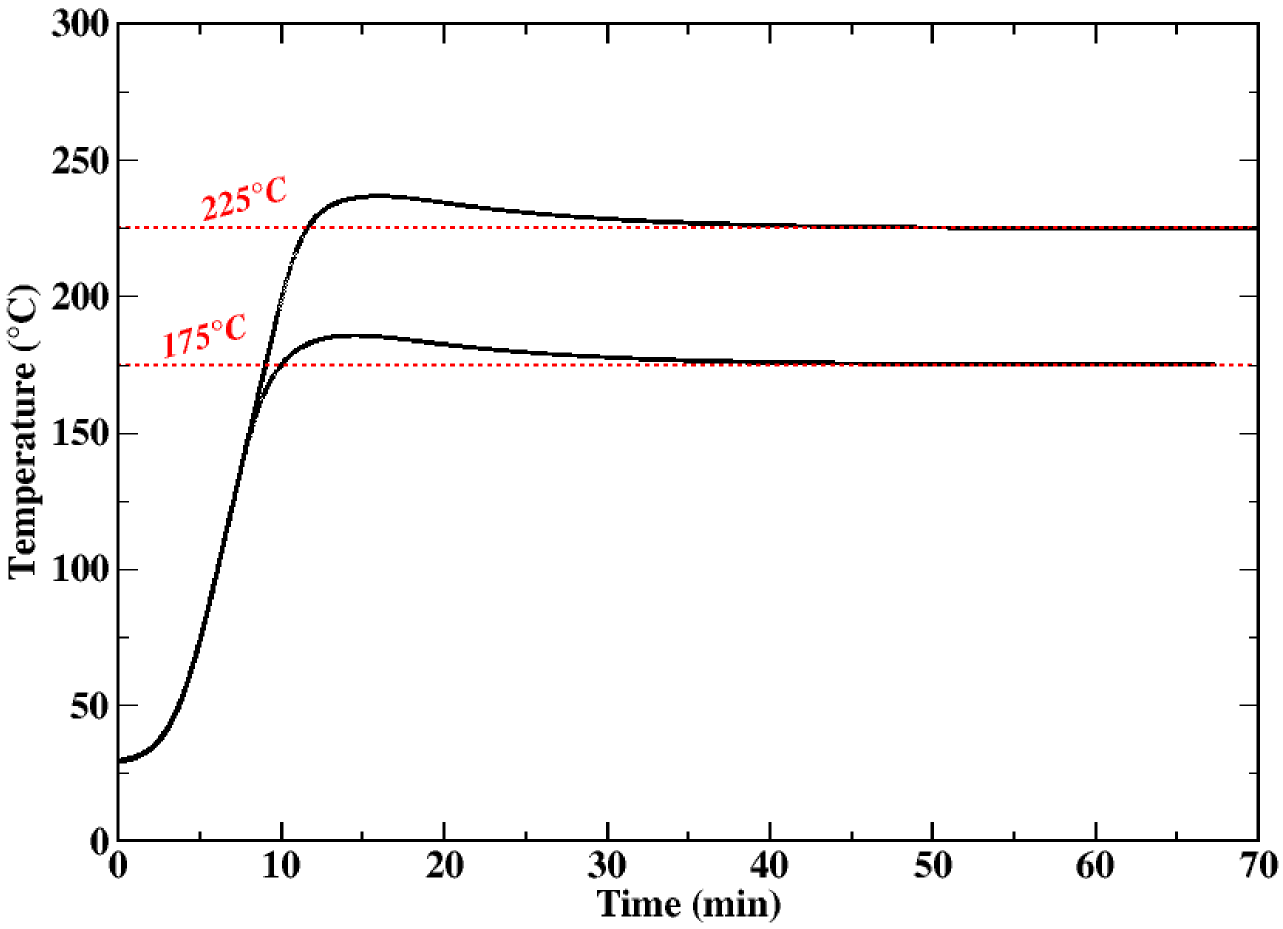

Figure S6. Temperature profile as a function of time during isothermal heating at 175°C and 225°C.
